# Diverse expression of TNF-α and CCL27 in serum and blister of Stevens–Johnson syndrome/toxic epidermal necrolysis

**DOI:** 10.1186/s13601-018-0199-6

**Published:** 2018-04-20

**Authors:** Fang Wang, Yanting Ye, Ze-Yu Luo, Qian Gao, Di-Qing Luo, Xingqi Zhang

**Affiliations:** 1 Department of Dermatology, First Affiliated Hospital, Sun Yat-sen University, No. 58, Zhongshan 2nd Road, Guangzhou, 510080 China; 2 Department of Dermatology, Eastern Hospital of First Affiliated Hospital, Sun Yat-sen University, 183 Huangpu Rd. E, Guangzhou, 510700 China

**Keywords:** Stevens–Johnson syndrome, Toxic epidermal necrolysis, Chemokine, Cutaneous T cell-attracting chemokine (CCL27), TNF-α

## Abstract

**Background:**

The pathogenesis of Stevens–Johnson syndrome (SJS)/toxic epidermal necrolysis (TEN) is not fully understood. Our previous study reported that chemokine CCL27 was overexpressed in serum of SJS/TEN patients. The objective of this study was to investigate the levels of CCL27 and TNF-α in serum and blister fluid from patients with SJS/TEN during the acute stage or resolution phase.

**Methods:**

A total of 27 patients with SJS/TEN and 39 healthy donors were recruited to the study. Serum and vesicular levels of CCL27 and TNF-α were determined by enzyme-linked immunosorbent assays.

**Results:**

Serum levels of CCL27 and TNF-α were significantly elevated in patients with SJS/TEN during the acute stage as compared to the resolution phase and also compared with levels observed in normal controls (*P* = 0.001/< 0.001; *P* = 0.012/< 0.001). Serum TNF-α levels were significantly higher in patients with SJS/TEN during the resolution phase compared with normal controls (*P* < 0.001). Serum CCL27 levels were positively correlated with TNF-α levels during the acute stage (*r*_*s*_ = 0.660; *P* < 0.001). Blister fluid exhibited much lower CCL27 levels than serum did during the acute stage (*P* = 0.008). TNF-α levels were much higher in vesicles in contrast to serum from acute stage (*P* = 0.040) as well as serum from resolution phase (*P* = 0.029).

**Conclusions:**

Our study demonstrated roles of CCL27 and TNF-α in promoting the course of SJS/TEN. CCL27 may act early in the course of disease, via the circulation, whereas TNF-α acts throughout the course of disease, in skin lesions.

## Background

Cutaneous adverse drug reactions (CADRs) are adverse drug reactions that occur on the skin. CADRs range from non-severe entities to lethal disorders. Stevens–Johnson syndrome (SJS)/toxic epidermal necrolysis (TEN) is the most severe life-threatening CADR, with a mortality rate of almost 25% [1]. SJS and TEN are considered to be the same entity with similar clinical-pathologic and immunologic features but varying degrees of severity [2]. SJS is the less severe form (skin is detached from less than 10% of the body surface area [BSA]), whereas TEN affects more than 30% of the BSA. Intermediate cases are classified as SJS/TEN overlap syndrome [3].

Although the pathogenesis of SJS/TEN is not fully understood, the skin-homing CD8^+^ T lymphocytes that express cutaneous lymphocyte antigen (CLA) have been shown to play a major role as effector cells [4–7]. After being activated, CD8^+^ T cell can launch various cytotoxic signals (including granulysin, perforin/granzyme B, Fas/Fas ligand, and cytokines/chemokines) to mediate disseminated keratinocyte death in skin lesions [8]. CCL27, also known as cutaneous T cell-attracting chemokine (CTACK), belongs to the CC chemokine family and its receptor is CCR10 [9]. CCL27 is reported to be expressed specifically on epithelial keratinocytes [9, 10]. CCL27 can selectively attract a subset of memory/effector T cells (both CD4^+^ and CD8^+^ T cells), positive for CLA and CCR10, from the peripheral blood to the targeted area of skin [11, 12]. CCL27 is highly upregulated in inflammatory skin conditions such as atopic dermatitis, psoriasis, and contact dermatitis [11]. In a study conducted by Tapia et al. [13], CCL27 was highly expressed in the skin lesions from two patients with SJS/TEN. Furthermore, the production of CCL27 can be augmented by tumor necrosis factor (TNF)-α in keratinocytes, through the activation of transcription factor NF-κB [14]. TNF-α is a pro-inflammatory cytokine that can be secreted by activated macrophages, natural killer cells and T cells [15]. TNF-α can induce cell apoptosis, cell activation, differentiation, and inflammatory processes [16, 17]. TNF-α is linked to the pathogenesis of SJS/TEN in apoptosis of keratinocytes and increased permeability of the vascular endothelium [18, 19].

In our previous study, we found that serum levels of CCL27 were significantly elevated in patients with SJS/TEN compared with normal controls [20]. The results indicated that CCL27 was involved in the pathogenesis of SJS/TEN. Compared with other chemokines (e.g., CXCL9, CXCL10) whose concentrations were elevated [21], CCL27 could correlate more tightly with detached BSA in patients with SJS/TEN. We sought to determine how CCL27 influences the dynamic course of SJS/TEN, as well as impacts on blister localization and the surrounding circulation. In this study, we measured levels of CCL27 and its related cytokine TNF-α in serum from patients with SJS/TEN during the acute stage and the resolution phase. We also measured levels of CCL27 in blisters, to study the exact role of CCL27 in the pathophysiology of SJS/TEN.

## Methods

### Patients and controls

A total of 27 patients with SJS/TEN were enrolled in the study during the period from January 2014 to June 2016. All patients were visiting the Department of Dermatology of First Affiliated Hospital, Sun Yat-sen University, Guangzhou, China, for treatment of their skin problems. The most probable culprit drug was identified according to the definition of adverse drug reactions provided by the World Health Organization [22]. The symptoms appeared at least 24 h after drug administration and improved upon discontinuation of the drug. The diagnosis of SJS/TEN was based on clinical manifestations: fever, involvement of mucous membranes, purpuric pricking macules or typical targets on face and trunk, positive Nikolsky sign, and extent of the epidermal detachment, as follows: SJS, detachment < 10% of BSA; TEN, detachment > 30% of BSA; SJS/TEN overlap, detachment between 10% and 30% of BSA. Patients enrolled in the study had recently been diagnosed and had not yet started any immunosuppressive or immunoregulatory agent to treat underlying diseases or drug-induced reaction. Patients who had positive serology for HIV, history of allergic disorders, malignant tumors, or rheumatoid diseases were excluded.

The acute stage was defined as the appearance of new macules, blisters or erosions, with positive Nikolsky sign. Resolution phase of the disorder was defined as a lack of new skin lesions, fading of old lesions, and negative Nikolsky sign. Patients were treated with < 0.4 mg/kg prednisone or equivalent dosage of a different steroid.

As controls, we selected 39 healthy adults, matched for age and gender. The controls had no history of drug hypersensitivity or cutaneous or immunological diseases. No patient was taking any medication at the time of inclusion in the study.

All procedures were approved by The Ethics Committee of First Affiliated Hospital of Sun Yat-sen University. Informed consent was obtained from all patients and normal controls.

### Sample collection

A total of 3 ml peripheral blood was obtained from each of 27 patients during the acute stage, before treatment. Samples were obtained from 39 normal individuals as well. Samples were obtained during the resolution phase from 18 patients. Fluid was taken from tense blisters during the acute stage by puncture aspiration into a syringe. Eight and six blister samples were collected to measure levels of CCL27 and TNF-α, respectively. Serum samples and blister fluid were obtained following centrifugation at 4 °C and centrifuged at 2500 rpm for 10 min, then stored at − 80 °C until testing.

### Measurement of CCL27 and TNF-α levels

Enzyme-linked immunosorbent assays (ELISAs) were performed in duplicate. CCL27 and TNF-α levels were detected using kits purchased from R&D Systems, Inc. (Minneapolis, MN, USA), and according to the manufacturer’s instructions.

### Statistical analysis

We analyzed the statistical distribution of cytokine levels and found that, except for serum TNF-α levels during the acute state in 27 SJS/TEN patients, cytokine levels were normally distributed. Data with normal distribution were described as mean ± standard deviation. For data not following a normal distribution, median (interquartile range) was used. Differences in cytokine levels were assessed with Student’s *t* test when they were normally distributed. For data that were not normally distributed, the Wilcoxon–Mann–Whitney nonparametric test was performed to detect significant differences. Correlation between cytokines was assessed using the Spearman rank correlation test. All tests were performed using IBM SPSS Statistics 18.0 software (SPSS Inc., Chicago, USA). Differences were considered significant at *P* < 0.05.

## Results

### Patient demographics and drugs implicated in SJS/TEN

The patient group comprised 23 SJS cases, 2 SJS/TEN overlap cases, and 2 TEN cases. The demographic and clinical characteristics of patients and controls are shown in Table 1. The culprit drugs identified most commonly were carbamazepine (n = 7, 25.9%) and allopurinol (n = 6, 22.2%).

**Table 1:** Demographic and clinical characteristics of patients and control group

Group	SJS/TEN			Controls
Subgroup	SJS	SJS/TEN overlap	TEN	
No.	23	2	2	39
Age (year), median (range)	43.0 (6–79)			42.2 (6–80)
Male/female (n)	17/10			25/14
Outcome (n)	A (23)	A (2)	A (2)	A (39)
Culprit drugs (n [%])				
Identified	22 (81.5)			
Carbamazepine	7 (25.9)			
Allopurinol	6 (22.2)			
Oxcarbazepine	2 (7.4)			
TCM	1 (3.7)			
Lamotrigine	1 (3.7)			
Amoxicillin	1 (3.7)			
Diazepam	1 (3.7)			
Tamsulosin	1 (3.7)			
NSAIDs	1 (3.7)			
Unidentified	5 (18.5)			

*A* alive, *NSAIDs* nonsteroidal anti-inflammatory drugs, *TCM* traditional Chinese medicine

### Serum levels of cytokines in patients and healthy subjects

**Fig. 1:**
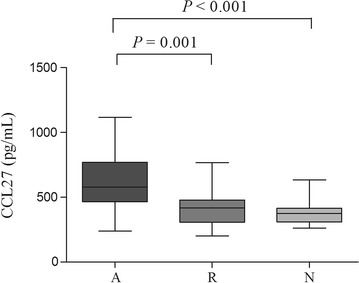
Serum levels of CCL27 from SJS/TEN patients during the acute phase (lane A), SJS/TEN patients during the resolution stage (lane R), and normal subjects (lane N). Statistical significance was determined by Student’s *t* test. Box = 25th and 75th percentiles; bars = min and max values; line inside box = median

CCL27 levels differed significantly among the three tested groups (*P* < 0.001). Higher levels of CCL27 were observed during the acute stage of SJS/TEN (629.2 ± 233.0 pg/ml) as compared with the resolution stage (421.9 ± 141.8 pg/ml; *P* = 0.001) or normal controls (377.2 ± 86.1 pg/ml; *P* < 0.001; Fig. 1). When samples obtained during the resolution stage were compared with those obtained from controls, no difference in CCL27 levels was found (*P* = 0.147).

**Fig. 2:**
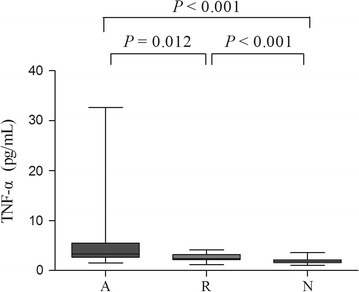
Serum levels of TNF-α from SJS/TEN patients during the acute phase (lane A), SJS/TEN patients during the resolution stage (lane R), and normal subjects (lane N). Statistical significance was determined by Wilcoxon-Mann–Whitney for comparisons between lane A and lane R, and between lane A and lane N. Student’s *t* test was used when comparing lane R with lane N. Box = 25th and 75th percentiles; bars = min and max values; line inside box = median

TNF-α levels also differed significantly among the groups (*P* < 0.001). Levels of TNF-α were elevated in patients with SJS/TEN during the acute stage (3.33 [2.80] pg/ml) compared with the resolution stage (2.53 ± 0.81 pg/ml; *P* = 0.012) or normal individuals (1.93 ± 0.49 pg/ml; *P* < 0.001; Fig. 2). Levels of TNF-α were also significantly higher in patients whose condition had resolved as compared to normal subjects (*P* < 0.001; Fig. 2).

**Fig. 3:**
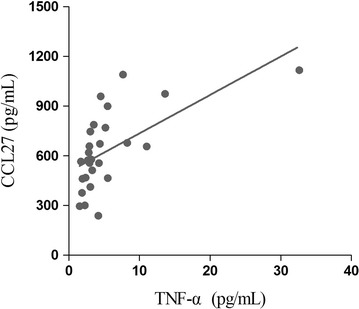
Positive correlation between serum CCL27 and TNF-α levels in patients with SJS/TEN, during the acute stage of disease (*r*_*s*_ = 0.660, *P* < 0.001). Statistical significance was determined by Spearman rank correlation test

During the acute stage of SJS/TEN, serum levels of CCL27 correlated positively with TNF-α levels (*r*_*s*_ = 0.660, *P* < 0.001; Fig. 3).

### Comparison of cytokine levels in serum and blister fluid

**Fig. 4:**
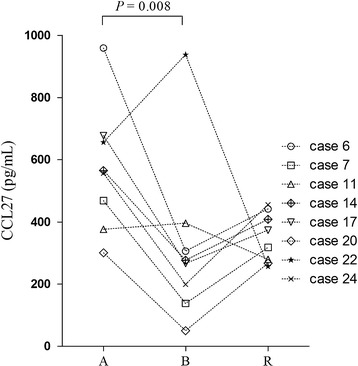
CCL27 levels in serum and blister fluid of patients with SJS/TEN. Each symbol represents the cytokine concentration from a patient. Case 7 and case 22 were the 2 patients with TEN. Statistical significance was determined by Student’s *t* test. *A* acute stage, *B* blister, *R* resolution phase

A total of 8 blister samples were obtained to test CCL27 levels. Average levels of CCL27 in the serum of acute-stage, blister samples, and resolution-phase serum from the 8 patients were 570.0 ± 203.9 pg/ml, 321.2 ± 270.7 pg/ml, and 350.0 ± 80.1 pg/ml, respectively. CCL27 levels differed significantly among groups (*P* = 0.043). CCL27 levels were much lower in blisters than in the serum obtained from patients during the acute stage of disease (*P* = 0.008; Fig. 4). The difference in CCL27 levels between blister and serum from the resolution phase was not significant (*P* = 0.799).

**Fig. 5:**
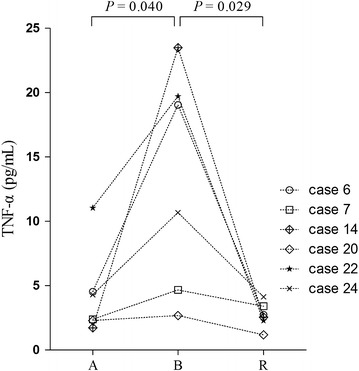
TNF-α levels in serum and blister fluid of patients with SJS/TEN. Each symbol represents the cytokine concentration from a patient. Statistical significance was determined by Student’s *t* test. *A* acute stage, *B* blister, *R* resolution phase

Blister fluid was taken from 6 patients with SJS/TEN to measure TNF-α levels; the average concentration was 13.4 ± 8.6 pg/ml. For all 6 patients, TNF-α levels in serum were also measured during the acute stage and resolution phase. TNF-α levels differed significantly among groups (*P* = 0.008). The blister fluid presented extremely high TNF-α levels; these levels were much higher than those measured in serum obtained during the acute stage (4.37 ± 3.46 pg/ml; *P* = 0.040) or resolution phase (2.71 ± 1.01 pg/ml; *P* = 0.029; Fig. 5).

## Discussion

Cytokines CCL27 and TNF-α are believed to play a vital role in the pathogenesis of SJS/TEN [13, 18]. In this study, we found dynamic changes in 2 cytokines during the course of the disorder, but their spatial distribution was quite different. These results suggested the two cytokines may act distinctly in the mechanisms of SJS/TEN.

In the present study, serum CCL27 levels were elevated during the acute stage of SJS/TEN but decreased to baseline when the disease improved. The overexpression of CCL27 in serum was consistent with our results reported previously [20]. Therefore, we strongly believe CCL27 is a factor promoting the course of SJS/TEN.

Strikingly, we discovered that CCL27 levels were much lower in blister fluid than in serum during the acute stage but close to serum levels during the resolution phase. To the best of our knowledge, very few reports have studied CCL27 levels in the blisters of SJS/TEN patients or have compared its levels in blister and serum samples from the same SJS/TEN patient. Our results could be the first to show an imbalance in CCL27 distribution between serum and blister in SJS/TEN. In previous studies, aside from high expression in epithelial keratinocytes, CCL27 levels were typically high in the superficial plexus of dermis during inflammatory processes such as atopic dermatitis, contact dermatitis, and healing of burn wounds [11, 23]. Through immunohistochemistry and measurements of mRNA expression, Tapia et al. [13] found CCL27 was highly expressed in the skin lesions of SJS/TEN patients. Based on these results, in combination with our findings, we hypothesized that CCL27 might have a very specific expression pattern in SJS/TEN: after being produced by keratinocytes, CCL27 is more likely to go “deep” into the circulation rather than being released to “superficial” blisters, in order to attract the effector T cells from circulation. Interestingly, in current study, a patient with TEN (case 22) presented blisters with extremely high CCL27 expression. As only two TEN patients were enrolled in the study, this discrepancy needs to be further explored in studies with large sample size.

It has been demonstrated that TNF-α plays a pivotal role in the pathogenesis of SJS/TEN [20]. Previous studies have reported increased levels of TNF-α in skin biopsy specimens or in blister fluid and serum [24–27]. Our results were consistent with previous studies. Because TNF-α levels were much higher in blister fluid than in serum in our study, and the overexpression of TNF-α derived from keratinocytes was observed by others [8], we believe TNF-α to be more active in targeted skin, and TNF-α may be linked to extensive skin detachment in SJS/TEN. This feature of TNF-α may relate to its important role in inflammation and its effect in inducing keratinocyte apoptosis through the caspase cascade and Fas/Fas ligand interaction [28].

Notably, in our study, serum TNF-α levels were higher in patients with SJS/TEN than in controls, even during the resolution phase. This suggested that TNF-α could act throughout the course of SJS/TEN. Compared with CCL27, TNF-α may exert a more prolonged effect. The findings provided experimental evidence for a therapeutic method of antagonizing the TNF-α pathway in treating patients with SJS/TEN [29–31].

The major limitations of this study include that we did not directly study CCL27 expression in epithelium, so that the distribution of CCL27 within skin lesions was not sufficiently reflected. In addition, the chemotaxis of CD8^+^ T cells mediated by CCL27 in SJS/TEN was not tested. In future experiments, we will carry out chemotaxis assays and perform in vivo tests to determine the precise role of CCL27 in SJS/TEN.

### Implications for research and practice

To date, data on the link between CCL27 and TNF-α in SJS/TEN are limited. Our study found a positive correlation between serum levels of CCL27 and TNF-α during the acute stage. Several studies have shown that TNF-α can induce keratinocytes to secrete CCL27 in vitro [13, 32]. Taking these findings into account, we hypothesized that the keratinocytes amplify inflammation in SJS/TEN. In that case, keratinocytes are not only the victims being attacked, but also the generators releasing more cytokines upon inflammatory stimulation. CCL27 produced by keratinocytes may serve as a target for treatment seeking to prevent SJS/TEN.

## Conclusions

In summary, our study demonstrated that cytokines CCL27 and TNF-α were of significant importance in promoting the course of SJS/TEN. However, their effects were not the same: CCL27 might act early and systemically, whereas, TNF-α may act more durably and locally.

## Abbreviations

BSA: body surface area
CADRs: cutaneous adverse drug reactions
CLA: cutaneous lymphocyte antigen
CTACK: cutaneous T cell-attracting chemokine
ELISAs: Enzyme-linked immunosorbent assays
NSAIDs: nonsteroidal anti-inflammatory drugs
SJS: Stevens–Johnson syndrome
TCM: traditional Chinese medicine
TNF-α: tumor necrosis factor-α
TEN: toxic epidermal necrolysis
